# Low-dose CT reconstruction via L1 dictionary learning regularization using iteratively reweighted least-squares

**DOI:** 10.1186/s12938-016-0193-y

**Published:** 2016-06-18

**Authors:** Cheng Zhang, Tao Zhang, Ming Li, Chengtao Peng, Zhaobang Liu, Jian Zheng

**Affiliations:** Suzhou Institute of Biomedical Engineering and Technology of Chinese Academy of Sciences, Suzhou, 215163 China; Changchun Institute of Optics, Fine Mechanics and Physics, Chinese Academy of Sciences, Changchun, 130033 China; University of Chinese Academy of Sciences, Beijing, 100049 China; Department of Electronic Science and technology, University of Science and Technology of China, Hefei, 230061 China

**Keywords:** Dictionary learning, Image reconstruction, L_1_-norm, Iteratively reweighted least squares

## Abstract

**Background:**

In order to reduce the radiation dose of CT (computed tomography), compressed sensing theory has been a hot topic since it provides the possibility of a high quality recovery from the sparse sampling data. Recently, the algorithm based on DL (dictionary learning) was developed to deal with the sparse CT reconstruction problem. However, the existing DL algorithm focuses on the minimization problem with the L_2_-norm regularization term, which leads to reconstruction quality deteriorating while the sampling rate declines further. Therefore, it is essential to improve the DL method to meet the demand of more dose reduction.

**Methods:**

In this paper, we replaced the L_2_-norm regularization term with the L_1_-norm one. It is expected that the proposed L_1_-DL method could alleviate the over-smoothing effect of the L_2_-minimization and reserve more image details. The proposed algorithm solves the L_1_-minimization problem by a weighting strategy, solving the new weighted L_2_-minimization problem based on IRLS (iteratively reweighted least squares).

**Results:**

Through the numerical simulation, the proposed algorithm is compared with the existing DL method (adaptive dictionary based statistical iterative reconstruction, ADSIR) and other two typical compressed sensing algorithms. It is revealed that the proposed algorithm is more accurate than the other algorithms especially when further reducing the sampling rate or increasing the noise.

**Conclusion:**

The proposed L_1_-DL algorithm can utilize more prior information of image sparsity than ADSIR. By transforming the L_2_-norm regularization term of ADSIR with the L_1_-norm one and solving the L_1_-minimization problem by IRLS strategy, L_1_-DL could reconstruct the image more exactly.

## Background

Nowadays, X-ray CT (computed tomography) is still one of the most important medical imaging technologies. Compared to other imaging methods, like ultrasonic imaging or magnetic resonance imaging, CT imaging has its own advantages to provide patients’ anatomical structure. CT images are of higher quality than the ultrasonic images. Compared to the magnetic resonance imaging, CT imaging is of faster imaging speed and CT images have a bit higher spatial resolution.

However, high quality CT images are now based on a noticeable X-ray radiation dose to the patient, which may result in a non-negligible lifetime risk of genetic or cancerous diseases [1]. This fact has become a major concern for clinical applications of CT scans. Therefore, this article focuses on reducing the radiation dose in CT and generating the clinically qualified image.

In order to reduce the radiation dose, one direct way is to lower mAs levels in CT data acquisition protocols. However, this approach will result in insufficient numbers of X-ray photons received by the detectors and hence increase the quantum noise level. This is a great challenge for these advanced methods which have taken the noise models into account. For example, PWLS (penalized weighted least-squares) based methods [2] can only deal with the noise-contaminated sinogram data to some extent. As a consequence, the radiation dose cannot be reduced evidently by this approach if the reconstructed images need to be qualified for clinical diagnosis. Another way to reduce imaging dose is to decrease the number of X-ray projections operated by fewer sampling angles. Yet, this will lead to serious streaking artifacts in the image reconstructed by the analytic-based algorithms like FBP (filtered backprojection algorithm) [3], since the analytic-based algorithms require that the number of projections should follow the Shannon/Nyquist sampling theorem [4].

To solve the under-sampled reconstruction problem, algebraic algorithms transform the problem to a series of linear equations and the reconstructed image is acquired by the iterative method. SART (simultaneous algebraic reconstruction technique) [5] is one of the typical algebraic iterative methods. But the images reconstructed by traditional algebraic algorithms do not satisfy the clinical image quality demand. Researchers are trying to improve the performance of the iterative reconstruction algorithms by introducing prior information of the reconstructed images to the reconstruction process.

Recently, the compressed sensing theory [6, 7] has been applied to the CT reconstruction problem. The reconstruction problem of the compressed sensing algorithm can be written as a constrained optimization

1 $$ \arg  {\mathop{{\rm min} }\limits_{{\boldsymbol{\upmu}}}} ({\mathbf{A}}{\boldsymbol{\upmu }} - {\hat{\mathbf{g}}})^{2} + \lambda R({\boldsymbol{\upmu}}) $$

where $({\bf A}{\boldsymbol{\upmu}} - {\hat{\mathbf{g}}})^{2}$ is the data fidelity term, **A** is the projection matrix modeling the forward projection, **μ** is the image vector to be reconstructed, ${\hat{\mathbf{g}}}$ is the projection vector, *R*(**μ**) is the regularization term including the prior information, *λ* is the regularization parameter adjusting the relative penalties on the regularization term and the data fidelity term. The regularization term is modeled by the prior information. One commonly used regularization term is the TV (total variation) norm, which is the sum of the absolute coefficients of the DGT (discrete gradient transform) of the reconstructed image. TV-based algorithm is usually used to solve the CT reconstruction problem since that most CT images are piecewise constant. So the TV norm is small enough to reflect the image sparsity. Algorithms like ASD-POCS (adaptive steepest descent projection onto convex sets) [8] and GPBB (gradient projection Barzilai Borwein) [9] are typical TV-based reconstruction algorithms. Besides the TV norm, the recent coming DL (dictionary learning) methods can generate the regularization term, which divide the CT image into many overlapped patches and calculate the sparse representations of the patches under the basis of an over-complete dictionary. While the TV-based methods take the image as a whole to measure the image sparsity, the DL methods have advantages over TV since they extract the sparse prior information from each overlapped image patch, which utilizes more sparse information than TV. Reference [10] combines the SIR (statistical iterative reconstruction) model with DL regularization term to deal with the low-dose reconstruction problem.

Although the compressed sensing algorithms behave well in the low-dose CT reconstruction problem, some low-contrast details of the reconstructed image are lost, especially when the sampling rate decreases further. In order to reserve more image information and satisfy the need of further radiation reduction, the TV-based algorithm developed a weighted TV regularization to help preserve the edge of the image [11]. When it comes to the DL-based algorithm, it is common that the regularization term is L_2_-norm error. Usually, algorithms based on L_2_-norm error minimization may lead to over-smoothing of the image, causing loss of detail. One way to alleviate this problem is to develop algorithms to minimize the L_1_-norm error. In this work, we develop a DL reconstruction algorithm with the L_1_-norm regularization term while the L_1_-minimizaiton problem is approximated by iteratively solving the weighted L_2_-minimization problem, known as IRLS (iteratively reweighted least squares) [12, 13]. The proposed L_1_-DL (L_1_ dictionary learning) algorithm is compared with ADSIR (adaptive dictionary based statistical iterative reconstruction), SART and GPBB to demonstrate the improvement of image quality based on the L_1_-norm regularization term.

The rest of the paper is organized as follows. In ‘‘Methods’’ section, firstly, the backgrounds of ADSIR and IRLS are reviewed. Then the L_1_-DL algorithm and its corresponding optimizing methods are described. After that, the workflow of the algorithm is provided. In section Simulation, a series of experiments are performed to demonstrate the proposed algorithm’s superiority. Finally, the section Conclusion with corresponding discussions and further analysis is provided.

## Methods

### Review of ADSIR

The previous work developed a DL based approach for low-dose X-ray CT with the statistical reconstruction model [10]. The related algorithm is reviewed in details as follows.

#### SIR model

Let *I* and *N* be integers and ${\mathbf{\mathbb{R}}}$ be the real space. By assuming a monochromatic source, measured data follow the Poisson distributionphantoms with full display

2 $$ y_{i} \sim {\text{Poisson}\{b_{i} e^{{-g_{i}}}   +\gamma_{i}}\},\quad i = 1, \ldots ,I $$

where ${\mathbf{b}} = (b_{1} ,b_{2} , \ldots ,b_{I} )^{T} \in {\mathbf{\mathbb{R}}}^{I \times 1}$ is the entrance X-ray intensity, ${\mathbf{y}} = (y_{1} ,y_{2} , \ldots ,y_{I} )^{T} \in {\mathbf{\mathbb{R}}}^{I \times 1}$ is the exit X-ray intensity, ${\mathbf{g}} = (g_{1} ,g_{2} , \ldots ,g_{I} )^{T} \in {\mathbf{\mathbb{R}}}^{I \times 1}$ is the integral of the linear attenuation coefficient with $g_{i} = [{\mathbf{A{\boldsymbol{\upmu}} }}]_{i} = \sum\nolimits_{j = 1}^{{N^{2} }} {a_{ij} \mu_{j} }$, ${\mathbf{A}} = \{ a_{ij} \} \in {\mathbf{\mathbb{R}}}^{{I \times N^{2} }}$ is the system matrix, the reconstructed image ${\varvec{\upmu}} = (\mu_{1} ,\mu_{2} , \ldots ,\mu_{{N^{2} }} )^{T}$ is a linear attenuation coefficient distribution, which transforms the initial image of *N* × *N* pixels to a vector ${\varvec{\upmu}} \in {\mathbf{\mathbb{R}}}^{{N^{2} \times 1}}$, *γ*_*i*_ represents the read-out noise.

The objective function of the SIR model is as

3 $$ \arg \mathop {\hbox{min} }\limits_{{\varvec{\upmu}}} \sum\limits_{i = 1}^{I} {\frac{{\omega_{i} }}{2}\left( {\left[ {{{\bf A}\boldsymbol{\upmu}}} \right]_{i} - \hat{g}_{i} } \right)^{2} } $$

where $\sum\nolimits_{i = 1}^{I} {(\omega_{i} /2)([{{\bf A}\boldsymbol{\upmu}}]_{i} - \hat{g}_{i} )^{2} }$ is the data fidelity term, ${\hat{\mathbf{g}}}= (\hat{g}_{1} ,\hat{g}_{2} , \ldots ,\hat{g}_{I} )^{T} \in {\mathbf{\mathbb{R}}}^{I \times 1}$ is the measured data of **g** calculated by $\hat{g}_{i} = \ln (b_{i} /(y_{i} - \gamma_{i} ))$, *ω*_*i*_ = (*y*_*i*_ − *γ*_*i*_)^2^/*y*_*i*_ is the statistical weight.

In the SIR model, the statistical weight reflects the confidence of the projection measurement along each path. The projection data through denser paths would have lower SNR (signal to noise ratios). Compared to the SART which minimizes a least square function, SIR deals with a statistically weighted least square function. However, this development is insufficient for the low-dose CT reconstruction that it is essential to introduce the regularization constraint.

#### DL model

Let *N*_0_ and *K* be integers. The DL regularization term is represented as

4 $$ R\left( {\varvec{\upmu}} \right) = \sum\limits_{s = 1}^{S} {||{\mathbf{E}}_{s} {\varvec{\upmu}} - {\boldsymbol{D\alpha}}_{s} ||_{2}^{2} } + \sum\limits_{s = 1}^{S} {{\boldsymbol\nu}_{s} ||{\varvec{\upalpha}}_{s} ||_{0} } $$

where ${\mathbf{E}}_{s} = \{ e_{nj}^{s} \} \in {\mathbf{\mathbb{R}}}^{{N_{o}^{2} \times N^{2} }}$ is an operator to extract patches with *N*_0_ × *N*_0_ pixels from the image, the image patches are overlapping. With a sliding distance of one pixel, the total number of the patches is *S* = (*N* − *N*_0_ + 1) × (*N* − *N*_0_ + 1). ${\mathbf{D}} = ({\mathbf{d}}_{1} ,{\mathbf{d}}_{2} , \ldots ,{\mathbf{d}}_{K} ) \in {\mathbf{\mathbb{R}}}^{{N_{0}^{2} \times K}}$ is the training dictionary, whose column ${\mathbf{d}}_{k} \in {\mathbf{\mathbb{R}}}^{{N_{0}^{2} \times 1}}$ is called an atom with the same size of a patch. Usually, the dictionary is redundant or over-complete (*N*_0_^2^ ≪ *K*). ${\varvec{\upalpha}}_{s} \in {\mathbf{\mathbb{R}}}^{K \times 1}$ has few nonzero components as a sparse representation of the patch by the dictionary basis **D**. *ν*_*s*_ is the regularization parameter different from *λ*.

#### ADSIR

By introducing the DL regularization term to the SIR model, the objective function of ADSIR is as

5 $$ \mathop {\hbox{min} }\limits_{{{{{\boldsymbol{\mu}} ,{\boldsymbol{\alpha}} ,{\mathbf{D}}}}}} \sum\limits_{i = 1}^{I} {\frac{{\omega_{i} }}{2}\left( {\left[ {{\mathbf{A{\boldsymbol{\upmu} }}}} \right]_{i} - \hat{g}_{i} } \right)^{2} } + \lambda \left( {\sum\limits_{s = 1}^{S} {||{\mathbf{E}}_{s} {\varvec{\upmu}} - {\mathbf{D{\boldsymbol\alpha }}}_{s} ||_{2}^{2} } + \sum\limits_{s = 1}^{S} {{\boldsymbol\nu}_{s} ||{\varvec{\upalpha}}_{s} ||_{0} } } \right) $$

Since **μ**, **α**_*s*_ and **D** are all unknown, the algorithm is iterated by the alternating minimization scheme, which divides the primary problem into two recursive steps—update of the dictionary model and update of the image. During each iteration process, keep the image **μ** unchanged firstly when the dictionary model is updated. And the objective function (5) becomes

6 $$ \mathop {\hbox{min} }\limits_{{{{\boldsymbol{\alpha} ,\mathbf{D}}}}} \sum\limits_{s = 1}^{S} {||{\mathbf{E}}_{s} {\varvec{\upmu}} - {\mathbf{D{\boldsymbol\alpha }}}_{s} ||_{2}^{2} } + \sum\limits_{s = 1}^{S} {\nu_{s} ||{\boldsymbol{\alpha}}_{s} ||_{0} } $$

which is the dictionary learning and sparse representation problem. The objective function (5) can be transformed to

$$ \begin{aligned}& \mathop {\hbox{min} }\limits_{{{{\boldsymbol{\alpha} ,\mathbf{D}}}}} \sum\limits_{s = 1}^{S} {||{\mathbf{E}}_{s} {\varvec{\upmu}} - {\mathbf{D\alpha }}_{s} ||_{2}^{2} }  \\ &||{\boldsymbol{\alpha}}_{s} ||_{0} \le L_{0}^{S}  \end{aligned} $$

where the sparse level *L*_0_^*S*^ is set as a fixed number, usually from 5 to 10. Then the L0-norm problem as (5) is transformed to the OMP question, which has no need to determine the value of *ν*_*s*_. The dictionary **D** is updated by the classic K-SVD (K Singular Value Decomposition) algorithm [14]. Then, the sparse representation **α**_*s*_ is updated by using the OMP (orthogonal matching pursuit) [15] algorithm based on recent dictionary. Once the dictionary model has been updated in the current iteration process, the image **μ** should be updated with **α**_*s*_ and **D** invariable. In other words, the problem transforms to the form as

7 $$ \mathop {\hbox{min} }\limits_{{\varvec{\upmu}}} \sum\limits_{i = 1}^{I} {\frac{{\omega_{i} }}{2}\left( {\left[ {{\mathbf{A{\boldsymbol{\upmu} }}}} \right]_{i} - \hat{g}_{i} } \right)^{2} } + \lambda \sum\limits_{s = 1}^{S} {||{\mathbf{E}}_{s} {\varvec{\upmu}} - {\mathbf{D{\boldsymbol\alpha }}}_{s} ||_{2}^{2} } $$

which consists of the data fidelity term $\sum\nolimits_{i = 1}^{I} {\omega_{i} ([{\mathbf{A{\boldsymbol{\upmu} }}}]_{i} - \hat{g}_{i} )^{2} /2}$ and the regularization term ∑ _*s*=1_^*S*^||**E**_*s*_**μ** − **Dα**_*s*_||_2_^2^. Since the regularization term is already a separable quadratic function. By replacing the data fidelity term with a separable paraboloid surrogate [16], the optimization can be iteratively solved by

8 $$ \begin{aligned} \mu_{j}^{t + 1} &= \left[ {\mu_{j}^{t} - \frac{{\sum\nolimits_{i = 1}^{I} {\left( {a_{ij} \omega_{i} \left( {\left[ {{\mathbf{A{\boldsymbol{\upmu} }}}^{t} } \right]_{i} - \hat{g}_{i} } \right)} \right) + 2\lambda \sum\nolimits_{s = 1}^{S} {\sum\nolimits_{n = 1}^{{N_{0}^{2} }} {e_{nj}^{s} \left( {\left[ {{\mathbf{E}}_{s} {\varvec{\upmu}}^{t} } \right]_{n} - \left[ {{\mathbf{D{\boldsymbol\alpha }}}_{s} } \right]_{n} } \right)} } } }}{{\sum\nolimits_{i = 1}^{I} {\left( {a_{ij} \omega_{i} \sum\nolimits_{k = 1}^{{N^{2} }} {a_{ik} } } \right) + 2\lambda \sum\nolimits_{s = 1}^{S} {\sum\nolimits_{n = 1}^{{N_{0}^{2} }} {e_{nj}^{s} \sum\nolimits_{k = 1}^{{N^{2} }} {e_{nk}^{s} } } } } }}} \right]_{ + } \\ &\quad j = 1,2, \ldots ,N^{2} \end{aligned} $$

#### IRLS

Consider a sparse signal *x* with length *N* (sparse means the signal has few nonzero components, that is ||*x*||_0_ ≪ *N*) is encoded by an *M* × *N* measurement matrix *Φ* with *M* < *N*, and the encoded signal is *y* = *Φx* with length *M*. Referred to [12, 13], the objective function with L_p_-norm minimization to solve the sparse signal is as

9 $$ \mathop {\hbox{min} }\limits_{x} ||x||_{p}^{p} ,{\kern 1pt} {\kern 1pt} {\kern 1pt} {\kern 1pt} {\text{subject}}{\kern 1pt} {\kern 1pt} {\kern 1pt} {\text{to}}{\kern 1pt} {\kern 1pt} {\kern 1pt} \rm\Phi x = y{\kern 1pt} {\kern 1pt} {\kern 1pt} {\kern 1pt} (0 < p \le 1) $$

IRLS can be used for solving (9) by replacing the L_p_-norm with a weighted L_2_-norm

10 $$ \mathop {\hbox{min} }\limits_{x} \sum\limits_{i = 1}^{N} {w_{i} x_{i}^{2} {\kern 1pt} {\kern 1pt} ,{\kern 1pt} {\kern 1pt} {\kern 1pt} {\kern 1pt} {\text{subject}}{\kern 1pt} {\kern 1pt} {\kern 1pt} {\text{to}}{\kern 1pt} {\kern 1pt} {\kern 1pt} \rm\Phi x = y} $$

where the weights are computed from previous iteration result *x*^(*t*−1)^. To make the L_2_-norm approximate to the L_p_-norm, the weights are calculated by

11 $$ w_{i} = \left|(x_{i}^{(t - 1)} )^{2} + \varepsilon \right|^{{\frac{p}{2} - 1}} $$

where a small *ɛ* > 0 is provided to ensure stability. Then the signal is iterated by

12 $$ x_{i}^{(t)} = Q_{t} \rm\Phi^{T} (\rm\Phi Q_{t} \rm\Phi^{T} )^{ - 1} y $$

where *Q*_*t*_ = *diag*(1/*w*_1_, 1/*w*_2_, …, 1/*w*_*n*_) is the diagonal matrix with entries $1/w_{i} = |(x_{i}^{(t - 1)} )^{2} + \varepsilon |^{{1 - \frac{p}{2}}}$

#### L_1_-DL

In the ADSIR model, the regularization term ∑ _*s*=1_^*S*^||**E**_*s*_**μ** − **Dα**_*s*_||_2_^2^ is the sum of the L_2_-norm of the difference between the image patch and its sparse representation. The L_2_-norm constraint tends to distribute the energy of ∑ _*s*=1_^*S*^||**E**_*s*_**μ** − **Dα**_*s*_||_2_^2^ to each image patch **E**_*s*_**μ** − **Dα**_*s*_uniformly. However, most CT images are piecewise constant, so that most image patches have small values of ||**E**_*s*_**μ** − **Dα**_*s*_||(equal or close to zero). A small part of the image patches have large values of ||**E**_*s*_**μ** − **Dα**_*s*_|| because they contain edge details and image information. In other word, the distribution of ||**E**_*s*_**μ** − **Dα**_*s*_||is sparse. So we propose the L_1_-DL method to make ||**E**_*s*_**μ** − **Dα**_*s*_|| converge to the sparse distribution by the L_1_-norm regularization term. The L_1_-DL utilizes more prior information of image sparsity (the sparse distribution of ||**E**_*s*_**μ** − **Dα**_*s*_||) than ADSIR.

Derived from the ADSIR algorithm, the objective function of the L_1_-DL method is generated by replacing the L_2_-norm of the regularization term with the L_1_-norm, which is

13 $$ \mathop {\hbox{min} }\limits_{{{\mathbf{{\boldsymbol{\upmu} ,\alpha ,D}}}}} \sum\limits_{i = 1}^{I} {\frac{{\omega_{i} }}{2}\left( {\left[ {{\mathbf{A{\boldsymbol{\upmu} }}}} \right]_{i} - \hat{g}_{i} } \right)^{2} } + \lambda \left( {\sum\limits_{s = 1}^{S} {||{\mathbf{E}}_{s} {\varvec{\upmu}} - {\mathbf{D\boldsymbol\alpha }}_{s} ||_{1} } + \sum\limits_{s = 1}^{S} {{\boldsymbol\nu}_{s} ||{\boldsymbol{\upalpha}}_{s} ||_{0} } } \right) $$

To make this optimization problem solvable, the patch-based weighted L_2_-norm similar to IRLS is introduced. Since the DL theory takes the sparse representation of the image patch as the sparse constraint, the weights are calculated by the information of each image patch, which is

14 $$ w_{s} = 1\left/\left[\left(\frac{1}{{N_{0}^{2} }}\sum\limits_{i = 1}^{{N_{0}^{2} }} {|[{\mathbf{E}}_{s} {\varvec{\upmu}}^{(t - 1)} ]_{i} - [{\mathbf{D}}^{(t - 1)} {\boldsymbol{\upalpha}}_{s}^{(t - 1)} ]_{i} |} \right) + \varepsilon \right]\right. $$

where *N*_0_^2^ is the dimension of **E**_*s*_**μ**^(*t*−1)^ and **D**^(*t*−1)^**α**_*s*_^(*−*1)^, the script (*t* − 1) means the corresponding terms are previous iteration results, a small *ɛ* > 0 is to ensure stability. The weights is not computed from each component of the vector **E**_*s*_**μ** − **Dα**_*s*_. We firstly calculate the average absolute value of the components of the vector **E**_*s*_**μ** − **Dα**_*s*_, and then take the reciprocal value of it as the weight of this patch. This strategy is different from the IRLS algorithm, which is in order to fit the nature of DL method that the image patch is treated as the basic unit.

By introducing the weights, the optimization problem becomes

15 $$ \mathop {\hbox{min} }\limits_{{{\mathbf{\boldsymbol{\upmu ,\upalpha ,D}}}}} \sum\limits_{i = 1}^{I} {\frac{{\omega_{i} }}{2}\left( {\left[ {{\mathbf{A{\boldsymbol{\upmu} }}}} \right]_{i} - \hat{g}_{i} } \right)^{2} } + \lambda \left( {\sum\limits_{s = 1}^{S} {w_{i} ||{\mathbf{E}}_{s} {\varvec{\upmu}} - {\mathbf{D{\boldsymbol\alpha }}}_{s} ||_{2}^{2} } + \sum\limits_{s = 1}^{S} {\nu_{s} ||{\boldsymbol{\upalpha}}_{s} ||_{0} } } \right) $$

The iteration is also operated by the alternating minimization scheme. When updating the dictionary model, the objective function (15) becomes

16 $$ \sum\limits_{s = 1}^{S} {w_{i} ||{\mathbf{E}}_{s} {\varvec{\upmu}} - {\mathbf{D{\boldsymbol\alpha }}}_{s} ||_{2}^{2} } + \sum\limits_{s = 1}^{S} {{\boldsymbol\nu}_{s} ||{\boldsymbol{\alpha}}_{s} ||_{0} } .$$

By the transformation $$ {\mathbf{E}}_{s} {\mathbf{{\boldsymbol{\upmu}}^{\prime}}} = \sqrt {w_{s} } {\mathbf{E}}_{s} {\varvec{\upmu}} , $$$$ {\mathbf{\boldsymbol\upalpha^{\prime}}}_{s} = \sqrt {w_{s} } {\varvec{\upalpha}}_{s} $$ and the equation $$ ||{\boldsymbol{\upalpha}}_{s} ||_{0} = ||\sqrt {w_{s} } {\varvec{\upalpha}}_{s} ||_{0}, $$ (16) can be transformed to

17 $$ \sum\limits_{s = 1}^{S} {||{\mathbf{E}}_{s} {\mathbf{\boldsymbol{\upmu}^{\prime}}} - {\mathbf{D\boldsymbol\upalpha^{\prime}}}_{s} ||_{2}^{2} } + \sum\limits_{s = 1}^{S} {\nu_{s} ||{\mathbf{\boldsymbol\upalpha^{\prime}}}_{s} ||_{0} } $$

which is as the same form as (6), so that the dictionary model can be updated by K-SVD and OMP.

When updating the image, the objective function (15) becomes

18 $$ \mathop {\hbox{min} }\limits_{{\varvec{\upmu}}} \sum\limits_{i = 1}^{I} {\frac{{\omega_{i} }}{2}\left( {\left[ {{\mathbf{A\boldsymbol{\upmu} }}} \right]_{i} - \hat{g}_{i} } \right)^{2} } + \lambda \sum\limits_{s = 1}^{S} {w_{s} ||{\mathbf{E}}_{s} {\varvec{\upmu}} - {\mathbf{D\boldsymbol\upalpha }}_{s} ||_{2}^{2} }. $$

Similar to (8), the formula to update the image is

19 $$ \begin{aligned} \mu_{j}^{t + 1} &= \left[ {\mu_{j}^{t} - \frac{{\sum\nolimits_{i = 1}^{I} {\left( {a_{ij} \omega_{i} \left( {\left[ {{\mathbf{A\boldsymbol{\upmu} }}^{t} } \right]_{i} - \hat{g}_{i} } \right)} \right) + 2\lambda \sum\nolimits_{s = 1}^{S} {w_{s} \sum\nolimits_{n = 1}^{{N_{0}^{2} }} {e_{nj}^{s} \left( {\left[ {{\mathbf{E}}_{s} {\varvec{\upmu}}^{t} } \right]_{n} - \left[ {{\mathbf{D\alpha }}_{s} } \right]_{n} } \right)} } } }}{{\sum\nolimits_{i = 1}^{I} {\left( {a_{ij} \omega_{i} \sum\nolimits_{k = 1}^{{N^{2} }} {a_{ik} } } \right) + 2\lambda \sum\nolimits_{s = 1}^{S} {w_{s} \sum\nolimits_{n = 1}^{{N_{0}^{2} }} {e_{nj}^{s} \sum\nolimits_{k = 1}^{{N^{2} }} {e_{nk}^{s} } } } } }}} \right]_{ + } \hfill \\& \quad j = 1,2, \ldots ,N^{2} \hfill \\ \end{aligned} $$

The convergence of L1-DL is much more difficult to prove, and is considered beyond the scope of this paper. However, our experimental results to be reported below seem suggesting the convergence of our proposed algorithms.

Above all, the workflow of the developed algorithm is exhibited in Algorithm I. In addition, the ordered subsets convex (OSC) algorithm [17] is utilized to accelerate the convergence. 

### Simulation results

**Fig. 1:**
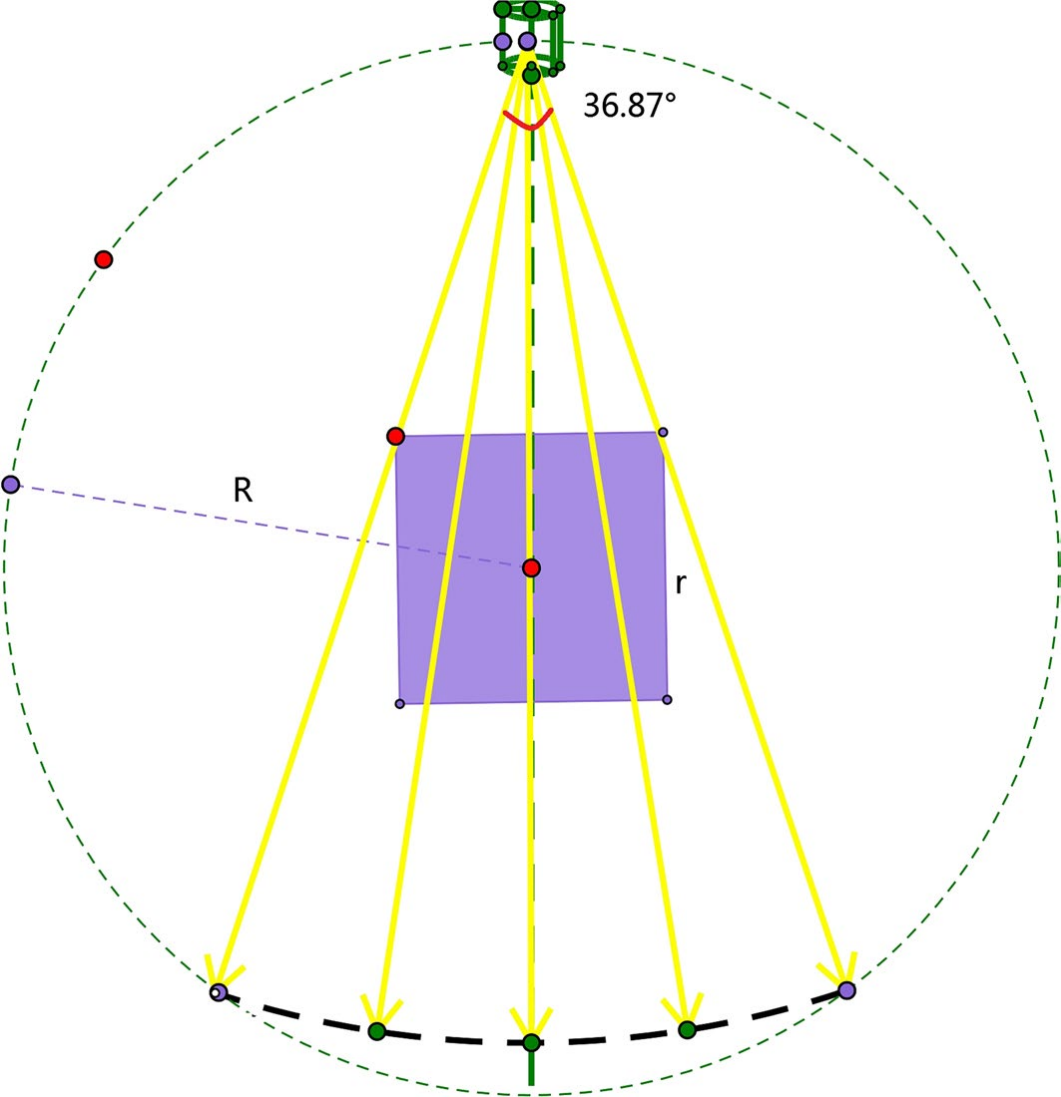
The scanning geometry

**Fig. 2:**
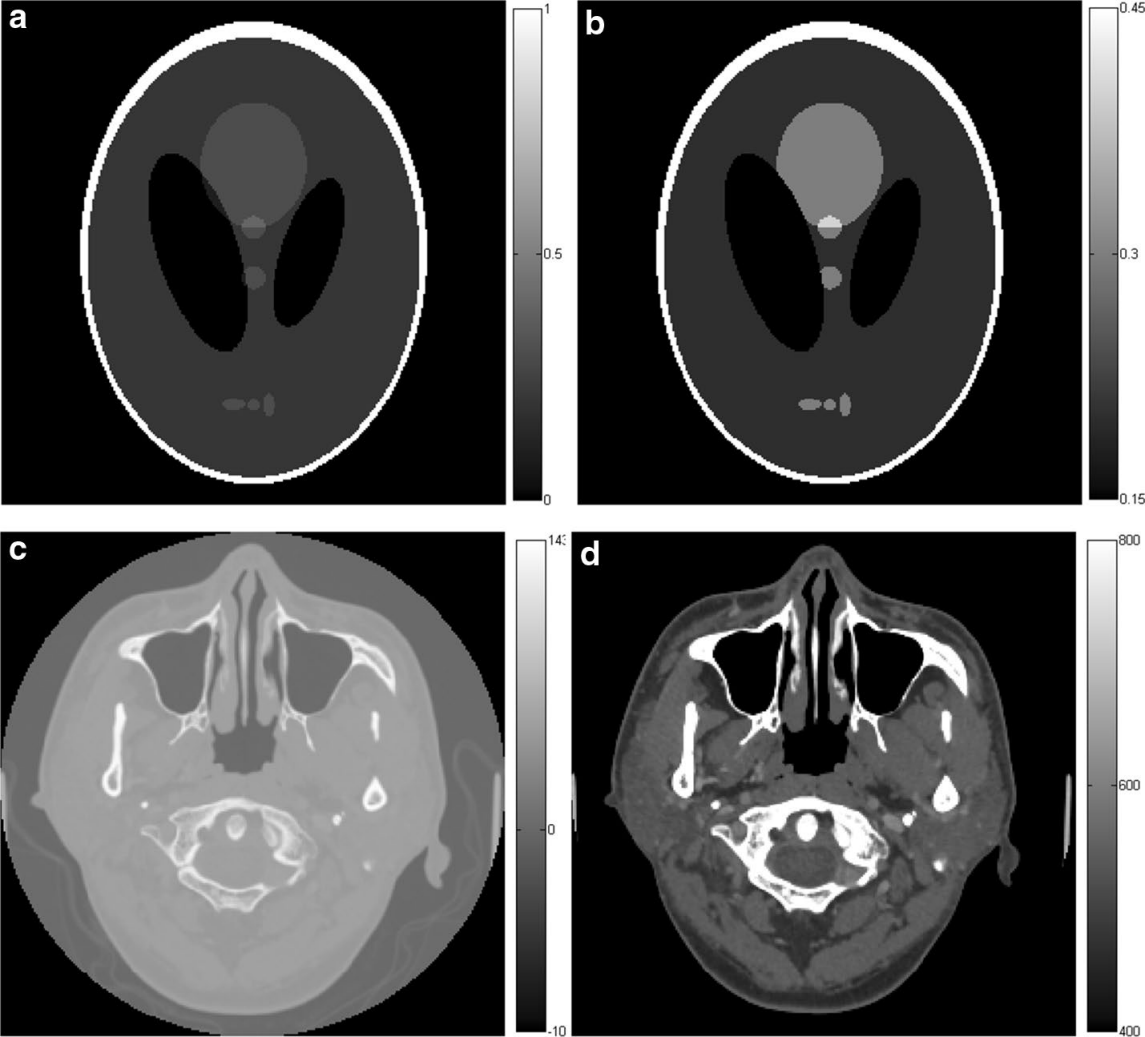
The simulation phantoms. The *first* to *third rows* refer to the Shepp–Logan phantom (**a**, **b**), and the human head slice (**c**, **d**), the *first column* refers to the phantoms with full display windows and the *second column* refers to the phantoms with proper window widths

To verify the effectiveness of the proposed L_1_-DL algorithm on low-dose CT reconstruction, several simulation experiments are designed. All the simulations are performed in MATLAB on a dual-core PC with 3.10 GHz Intel Core i5-2400. The proposed algorithm is compared with SART, GPBB and ADSIR. The scanning geometry is the fan-beam geometry shown in Fig. 1. The size of the phantom is *r* = 20 cm, the radius of the scanning circle (or the distance from the radiation source to the central point of the phantom) is *R* = 40 cm. For each projection view, 512 detector elements were equi-angularly distributed with the field angle being 36.87°. The distance from the radiation source to the detector elements is 75.895 cm. During the simulation, the scanning circle covers 360° around the imaging phantom, and the size of the reconstructed image is 256 × 256 pixels. The phantoms under simulations are respectively the Shepp–Logan phantom, and the human head slice from clinic, which are shown in Fig. 2 with full and part display window. The biomedical images are often observed by a proper window width to find more details. The number of different densities in the Shepp–Logan phantom is 6, and we set the density of water as 0.2.

Some details about the proposed algorithm (like the setting of some variables) are explained as follows. The image patches are overlapping with a sliding distance of one pixel, and the patches are with *N*_0_ × *N*_0_ pixels. The dictionary is consisted of *K* atoms. When updating the dictionary by K-SVD algorithm, the sparsity level is *L*_0_^*D*^. The sparsity level of the sparse representation **α**_*s*_ is set as *L*_0_^*S*^. The iterative stopping criterions are *ɛ*_1_ and *ɛ*_2_. The values of all the corresponding variables are provided in Table 1. In the image processing field, *K* = 4 × *N*_0_ × *N*_0_ is a conventional choice to ensure the redundancy of the dictionary. The patch size also influences the quality of the image. If the patch size is too small, it could not effectively catch features in an image. On the other hand, a larger patch size may lead to an over-smoothed image and corresponds to a larger number of atoms in a dictionary, which would increase the computational cost. For image processing, *N*_0_ = 8 is a proper value with considering both image quality and computational cost [18]. The sparsity level *L*_0_^*D*^ and *L*_0_^*S*^ are empirically determined according to the complexity of the image to be reconstructed.

**Table 1:** Summary of the parameter selections

No.	Variable	Meaning	Value
1	*N*_0_	Scale of the patch	8
2	*K*	Number of atoms in the dictionary	256
3	*L*_0_^*D*^	Sparsity level for dictionary learning	5
4	*L*_0_^*S*^	Sparsity level for sparse representation	5
5	*ɛ*_1_	Stopping criterion about the projection	0.001
6	*ɛ*_2_	Stopping criterion about the sparse representation	0.001

In addition, all the experiments are simulated by photon number counting method. To perform the OSC algorithm, the transmission data can be calculated with $y_{i} = b_{i} e^{{ - \hat{g}_{i} }} = e^{{ - \hat{g}_{i} }}$ by setting the entrance X-ray intensity *b*_*i*_ as the number of photons. L1-DL and ADSIR set the initial guess of the image as a random matrix while GPBB and SART set all the values of the elements of the initial matrix as 1. OSC is utilized to accelerate the convergence of L1-DL and ADSIR, with 10 subsets. The number of iterations of OSC is 30. GPBB and SART are not accelerated by OSC. GPBB and SART stop the iteration when the number of iterations reaches 1000. ADSIR stops when the conditions below are met at the same time:

$$\left\{
\begin{array}{ll}
\frac{|\delta^{(t)} - \delta^{(t - 1)} |}{\delta^{(t)} } <
\varepsilon_{1} ,&\quad \delta^{(t)} = \sum\nolimits_{i = 1}^{I}
\frac{\omega_{i}}{2}\left( {\left[ {\mathbf{A
}}{\boldsymbol{\upmu}}^{(t)}  \right]_{i} - \hat{g}_{i} }\right)^{2}
 \\ \frac{|\eta^{(t)} - \eta^{(t - 1)} |}{\eta^{(t)} } <
\varepsilon_{2} , &\quad \eta^{(t)} = \sum\nolimits_{s = 1}^{S}
||{\mathbf{E}}_{s} {\boldsymbol{\upmu}}^{(t - 1)} - {\mathbf{D}}^{(t)}
{\boldsymbol{\upalpha}}_{s}^{(t)} ||_{2}^{2}
\end{array} \right.$$

### Reconstruction of different sparse levels

In this simulation, the forward simulation and inverse reconstruction are all performed in 2D. During the simulation, the scanning circle covers 360° range around the phantom. The scanning step of tomographic angels of the Shepp–Logan phantom is set as 3°(120 views) and 6°(60 views) respectively. The scanning step of tomographic angels of the human head slice slice is set to 2°(180 views) and 4°(90 views) respectively.

We choose the SART, GPBB, and ADSIR algorithms to be the comparisons besides our proposed L_1_-DL algorithm. The regularization parameter of GPBB is chosen by tests (from 0.1 to 2, the length of step is 0.1). We choose the best one to perform GPBB. When it comes to the DL methods, a proper selection of the regularization parameter *λ* is a vital problem. A bigger *λ* weakens the effect of the data fidelity term, generating a loss of some fine details in the image while a smaller *λ* weakens the effect of the regularization term as the sparse constraint, resulting in more noise and streak artifacts in the reconstructed image. In this article, the regularization parameter of ADSIR is determined by a similar model as Ref. [19] creates. The model firstly reconstructs the image by setting *λ* as infinite, then calculates the difference between the forward projection and the scanning data. After that, the proper value of *λ* can be calculated by a fitting function based on the difference. In the proposed method (L_1_-DL), the weights introduced to the algorithm would influence the regularization parameter. To eliminate this effect, we multiply the weights with a constant which is the average value of $(1/N_{0}^{2} )\sum\nolimits_{i = 1}^{{N_{0}^{2} }} {|[{\mathbf{E}}_{s} {\varvec{\upmu}}^{(t - 1)} ]_{i} - [{\mathbf{D}}^{(t - 1)} {\varvec{\upalpha}}_{s}^{(t - 1)} ]_{i} |}$. The weights are calculated by

20 $$ \begin{aligned} w_{s} &= C\left/ \left[\left(\frac{1}{{N_{0}^{2} }}\sum\nolimits_{i = 1}^{{N_{0}^{2} }} {|[{\mathbf{E}}_{s} {\varvec{\upmu}}^{(t - 1)} ]_{i} - [{\mathbf{D}}^{(t - 1)} {\boldsymbol{\alpha}}_{s}^{(t - 1)} ]_{i} |} \right) + \varepsilon \right]\right.\\ C &= \frac{1}{S}\sum\limits_{s = 1}^{S} {\frac{1}{{N_{0}^{2} }}\sum\limits_{i = 1}^{{N_{0}^{2} }} {|[{\mathbf{E}}_{s} {\varvec{\upmu}}^{(t - 1)} ]_{i} - [{\mathbf{D}}^{(t - 1)} {\boldsymbol{\alpha}}_{s}^{(t - 1)} ]_{i} |} } \end{aligned} $$

With this modification, we select the regularization parameter of L_1_-DL as the same as the one of ADSIR. The regularization parameters of different phantom simulations are shown in Table 2. The rationality and influence of the regularization parameter selection will be discussed later.

**Table 2:** Selections of the regularization parameters with different sampling rates

	Shepp–Logan		Head slice	
	120 veiws	60 views	180 views	90 views
DL methods	3.831 × 10^3^	3.823 × 10^3^	380.7	380.1
GPBB	0.2	0.4	0.2	0.5

**Fig. 3:**
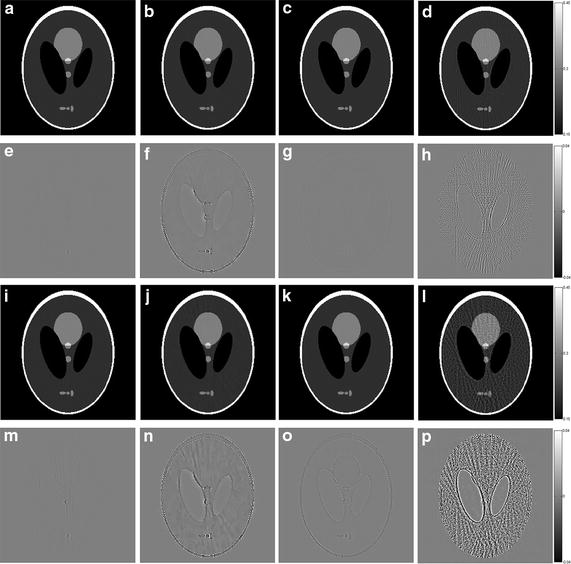
The reconstruction results of the Shepp–Logan phantom. **a**–**d** The image reconstructed by L1-DL, ADSIR, GPBB and SART respectively with 120 scanning views data; **e**–**h** the difference between the reconstructed image (**a**–**d**) and the original image (OI); **i**–**l** the image reconstructed by L1-DL, ADSIR, GPBB and SART respectively with 60 scanning views data; **m**–**p** the difference between the reconstructed image (**i**–**l**) and the original image (OI)

**Fig. 4:**
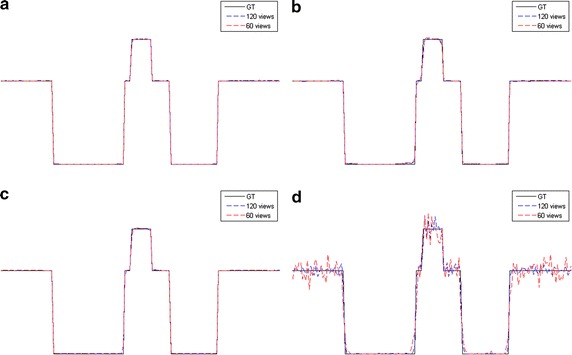
The horizontal intensity profiles through the center of the original and reconstructed images of the Shepp–Logan phantom. **a** L1-DL; **b** ADSIR; **c** GPBB; **d** SART

**Fig. 5:**
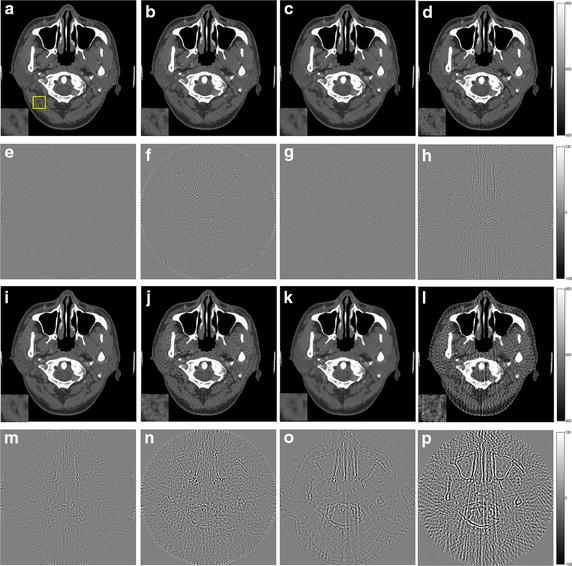
The reconstruction results of the human head slice. **a**–**d** The image reconstructed by L1-DL, ADSIR, GPBB and SART respectively with 180 scanning views data; **e**–**h** the difference between the reconstructed image (**a**–**d**) and the original image (OI); **i**–**l** the image reconstructed by L1-DL, ADSIR, GPBB and SART respectively with 90 scanning views data; **m**–**p** the difference between the reconstructed image (**i**–**l**) and the original image (OI)

**Fig. 6:**
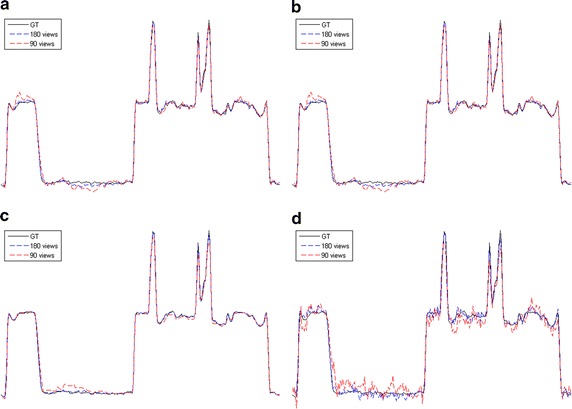
The vertical intensity profiles through the center of the original and reconstructed images of the human head slice. **a** L1-DL; **b** ADSIR; **c** GPBB; **d** SART

The reconstruction results of the simulations by using these four algorithms are shown in Figs. 3, 4 (Shepp–Logan phantom) and Figs. 5, 6 (human head slice). The negative values in reconstructed image during the iterative process are set to be zero. The results indicate the density of the phantoms. The images of human head slice are displayed by transforming the density to the CT attenuation value. The CT value is calculated by

21 $$ {\text{CT}}{\kern 1pt} {\kern 1pt} {\text{value = }}\frac{{\mu - \mu_{\text{water}} }}{{\mu_{\text{water}} }} \times 1000 $$

where *μ*_water_ is the linear attenuation coefficient of water.

By taking the original phantom as a gold standard to provide a numeric quantification of the results, the RMSE (root mean square error) of the reconstructed images is introduced to measure the difference between the reconstructed image and the original image in L_2_-norm. This criterion is defined as:

22 $$ {\text{RMSE}} = \sqrt {\frac{{\sum\nolimits_{i,j} {\left( {\mu_{ij} - \mu_{ij}^{truth} } \right)^{2} } }}{{N^{2} }}} $$

where *μ*_*ij*_^*truth*^ means the gray-value of the original image. The unit of RMSE can be HU by transform the linear attenuation coefficient to CT value according to Eq. (21). It is easy to demonstrate that the smaller the value of RMSE is, the better quality of the image is. The quantitative results are shown in Table 3.

**Table 3:** RMSEs (HU) of reconstructed images of different sampling rates

Phantom	L_1_-DL	ADSIR	GPBB	SART
Shepp–Logan				
120 views	1.647	22.62	2.658	34.73
60 views	2.867	31.72	11.04	94.62
Head slice				
180 views	6.228	15.50	4.282	17.87
90 views	10.28	25.55	14.78	41.42

In Figs. 3 and 5, all the reconstructed images of the same phantom are shown in the same proper display window. In Fig. 3, the results of SART are the worst among the four algorithms. Even with 120 sampling views, the result is ruined by the streak artifacts, which deteriorates seriously when the sampling rate decline to half. The reason for the bad results of SART is that this algorithm is a simple iterative algorithm that has no regularization term to utilize the prior information about the reconstructed image. Compared to SART, GPBB and ADSIR perform better with help of the regularization term utilizing the sparse constraint. Figure 3f, n contain some edge structures, which proves the over-smoothing effect of ADSIR. When projection views reduce to 60, the image reconstructed by ADSIR is influenced by some artifacts. L_1_-DL and GPBB perform well in the 120 views situation. The RMSE of these two methods are 1.647HU and 2.658HU respectively, which are both tiny. And Fig. 3e, g reveal that the difference is hard to recognize. When the sampling views reduce, tiny edge structures emerge in Fig. 3o and the RMSE of GPBB increases to 11.04. Figure 4 display the horizontal intensity profiles through the center of reconstructed images compared to the original image. It is shown that the effects of the reduction of the sampling views are arranged as: SART > ADSIR > GPBB > L_1_-DL. In Table 3, the RMSE of L_1_-DL under 60 views situation is much bigger than the RMSEs of ADSIR and SART with 120 views situation, which certifies that the proposed algorithm can adapt to further radiation dose reduction Table 4.

**Table 4:** Selections of the regularization parameters with different noise levels

	Shepp–Logan		Head slice	
	60 views 2 million photons	60 views 1 million photons	180 views 2 million photons	90 views 2 million photons
DL methods	4.841 × 10^3^	8.141 × 10^3^	1.451 × 10^3^	1.725 × 10^3^
GPBB	0.4	0.5	0.5	0.6

When it comes to the human head slice in Figs. 5 and 6, the results are similar to the Shepp–Logan phantom. But the original image of the human head slice is more complex than the Shepp–Logan phantom, and is close to the real reconstruction process of the biomedical images. Although the RMSE of the proposed algorithm is a little bigger than GPBB in 180 views situation, L_1_-DL is better than GPBB when the number of views reduces to 90. In 90 views situation, the image of GPBB, displayed as Fig. 5k, has some artifacts and the spatial resolution is worse than L_1_-DL by checking the enlarged region of the images. In addition, the RMSEs of the proposed algorithm is much better than the ones of ADSIR. Considering the proposed algorithm is modified based on the reconstruction model of ADSIR, the improvement of image quality is obvious.

### Robustness to the noise

In the practical applications, the measurements of the radiation projections are usually polluted by noise, which demands that the algorithm should be robust to the noise. To evaluate the tolerance to noise of the proposed algorithm and other three ones, we choose to add some Poisson noise to the projection data for test. The scanning step of tomographic angels of the Shepp–Logan phantom is set to 6°(60 views) and the simulation numbers of photons emitting from the X-ray source to each detector are 2 million and 1 million. The detected numbers of photons are polluted by Poisson noise. The scanning step of the human head slice is set to 2°(180 views) and 4°(90 views) respectively. The simulation number of photons is 2 million.

**Fig. 7:**
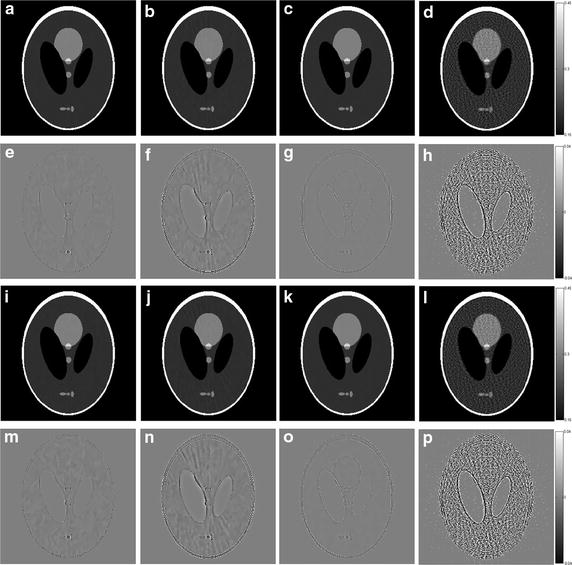
The reconstruction results of the Shepp–Logan phantom. **a**–**d** The image reconstructed by L1-DL, ADSIR, GPBB and SART respectively with 60 scanning views data simulated by 2 million photons; **e**–**h** the difference between the reconstructed image (**a**–**d**) and the original image (OI); **i**–**l** the image reconstructed by L1-DL, ADSIR, GPBB and SART respectively with 60 scanning views data simulated by 1 million photons; **m**–**p** the difference between the reconstructed image (**i**–**l**) and the original image (OI)

**Fig. 8:**
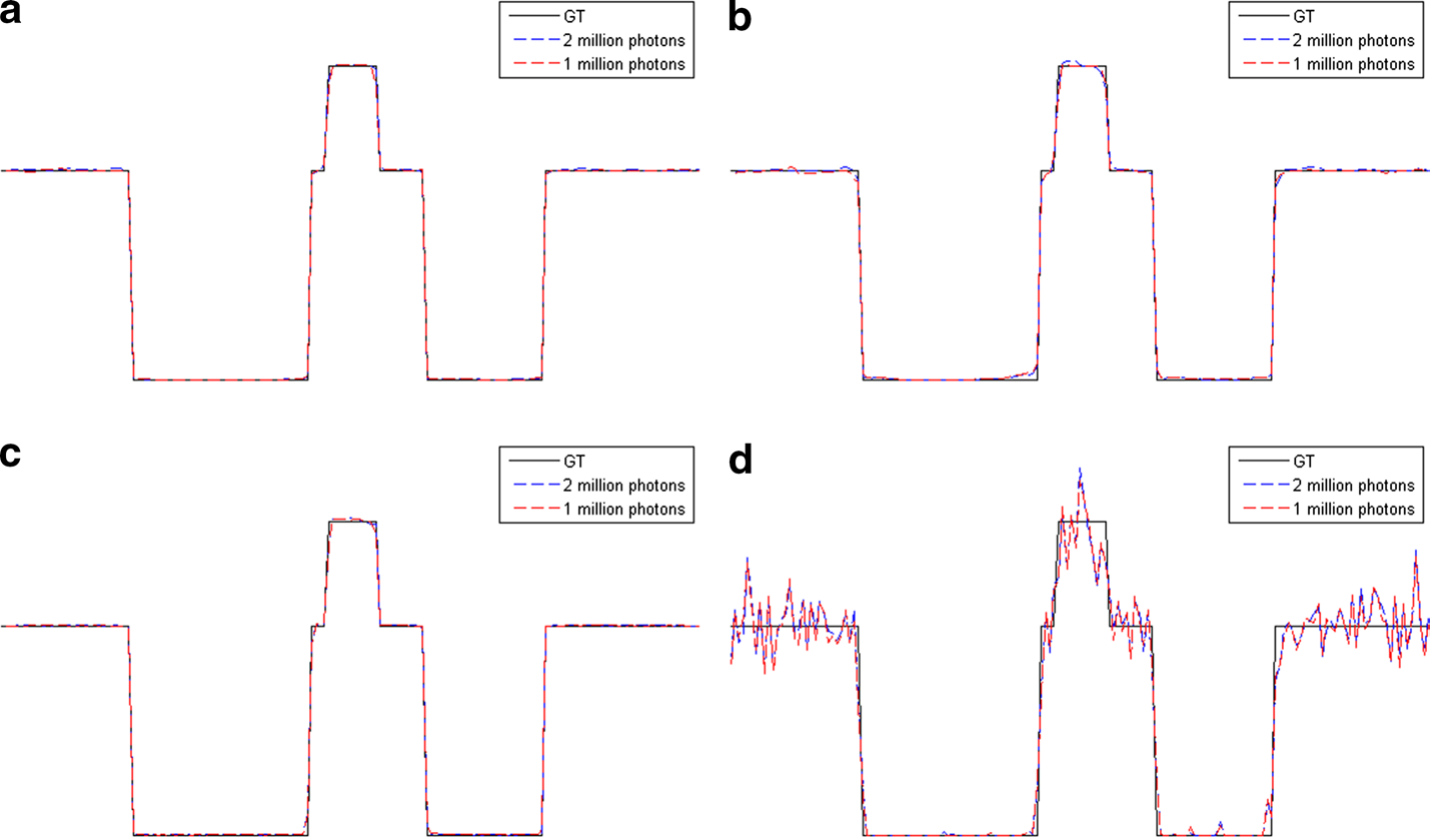
The horizontal intensity profiles through the center of the original and reconstructed images of the Shepp–Logan phantom with Poisson noise polluted projection data. **a** L1-DL; **b** ADSIR; **c** GPBB; **d** SART

**Fig. 9:**
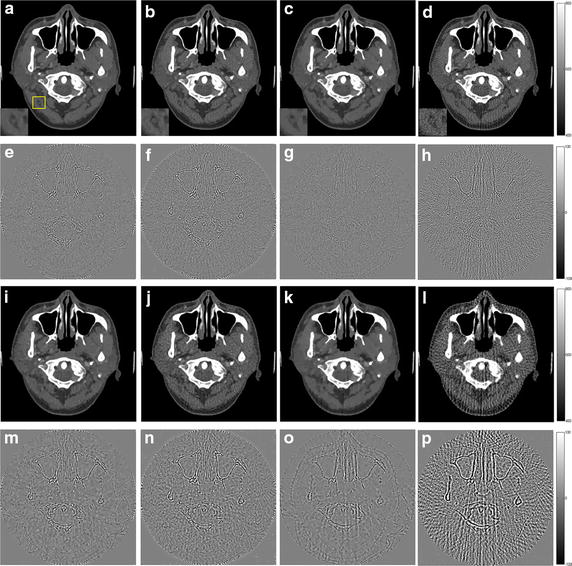
The reconstruction results of the human head slice. **a**–**d** The image reconstructed by L1-DL, ADSIR, GPBB and SART respectively with 180 scanning views data simulated by 2 million photons; **e**–**h** the difference between the reconstructed image (**a**–**d**) and the original image (OI); (**i**–**l**) the image reconstructed by L1-DL, ADSIR, GPBB and SART respectively with 90 scanning views data simulated by 2 million photons; **m**–**p** the difference between the reconstructed image (**i**–**l**) and the original image (OI)

**Fig. 10:**
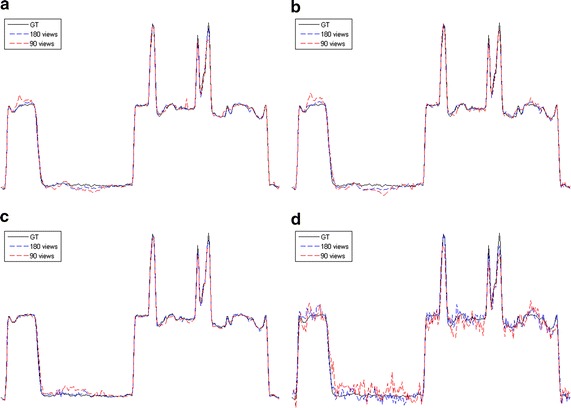
The vertical intensity profiles through the center of the original and reconstructed images of the human head slice with Poisson noise polluted projection data. **a** L1-DL; **b** ADSIR; **c** GPBB; **d** SART

The reconstructed images of the simulations are shown in Figs. 7, 8, 9, 10 and the RMSEs are provided in Table 5. Compared to the results with no noise polluted in Figs. 3, 4, 5 and 6, the image quality of SART degrades fast with the noise level increasing while GPBB, L_1_-DL and ADSIR are more robust to the noise. In the Shepp–Logan experiments, L_1_-DL is better than GPBB and ADSIR comparing the RMSEs. The difference between the reconstructed image and original image shown in Fig. 7 indicates the image quality is arranged as: L_1_-DL > GPBB > ADSIR > SART. In the head slice simulations, the RMSE of SART is a bit smaller than ADSIR, but the image reconstructed by SART are ruined by noise and artifacts. When it comes to L_1_-DL and GPBB, the RMSE of GPBB is better than L_1_-DL in 180 views situation, and the RMSE of these two methods are same in 90 views situation. The results (Fig. 9i–l) of the 90 views simulation with noise indicate that all the four algorithms lose the structure in the yellow rectangle region. L_1_-DL is still better than ADSIR and has a bit higher spatial resolution than GPBB.

**Table 5:** RMSEs (HU) of reconstructed images of different noise levels

Phantom	L_1_-DL	ADSIR	GPBB	SART
Shepp–Logan				
60 views, 2 million photons	10.87	31.3715	14.78	139.4
60 views, 1 million photons	11.68	34.84	15.43	139.9
Head slice				
180 views, 2 million photons	13.46	24.81	9.582	22.86
90 views, 2 million photons	16.83	30.54	16.83	44.49

### Convergence rate

**Fig. 11:**
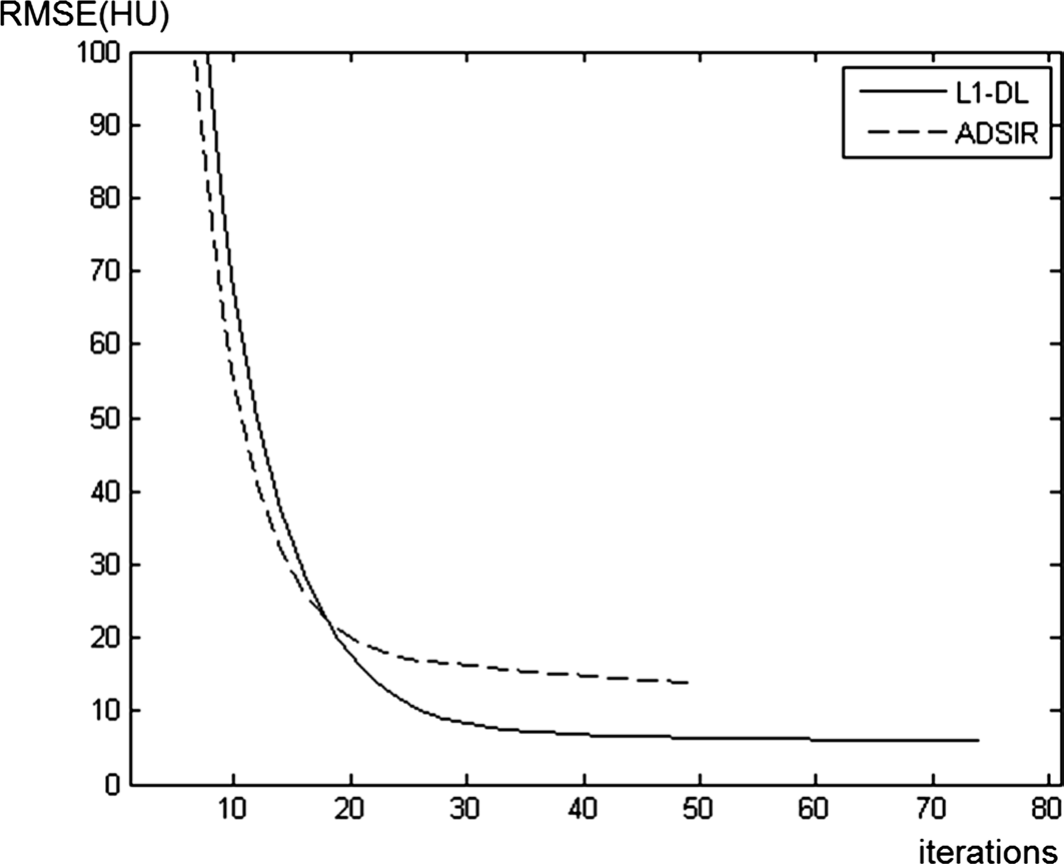
The RMSE as function of the number of iterations depending on the iterative image reconstructed by L1-DL and ADSIR

To explain the convergence rate of L_1_-DL compared to ADSIR, the RMSEs of the images reconstructed by these two algorithms are shown as functions of the number of iterations in Fig. 11. The projection data are simulated on the human head slice with 180 scanning views, which is not polluted by noise. L_1_-DL stops at the 72th iteration while ADSIR stops at the 49th iteration. Since the iterative process of L_1_-DL only has one additional step, which is updating the weight function, it takes almost the same time as ADSIR to perform one iteration. The times consumed by one iteration of ADSIR and L_1_-DL are 89 and 92 s respectively. By checking the first 20 iterations, it can be found that the RMSE decreasing rate of ADSIR is only a bit faster than L_1_-DL at first. So it is claimed that L_1_-DL needs more iterations than ADSIR for convergence because of the better reconstructed result, but the convergence rate of the two algorithms are almost the same. However, it takes several tens of minutes for both ADSIR and L_1_-DL to reach the stopping points, so that some accelerating methods and high computation efficiency are the expectations for the real time imaging.

### Regularization parameter investigation

**Fig. 12:**
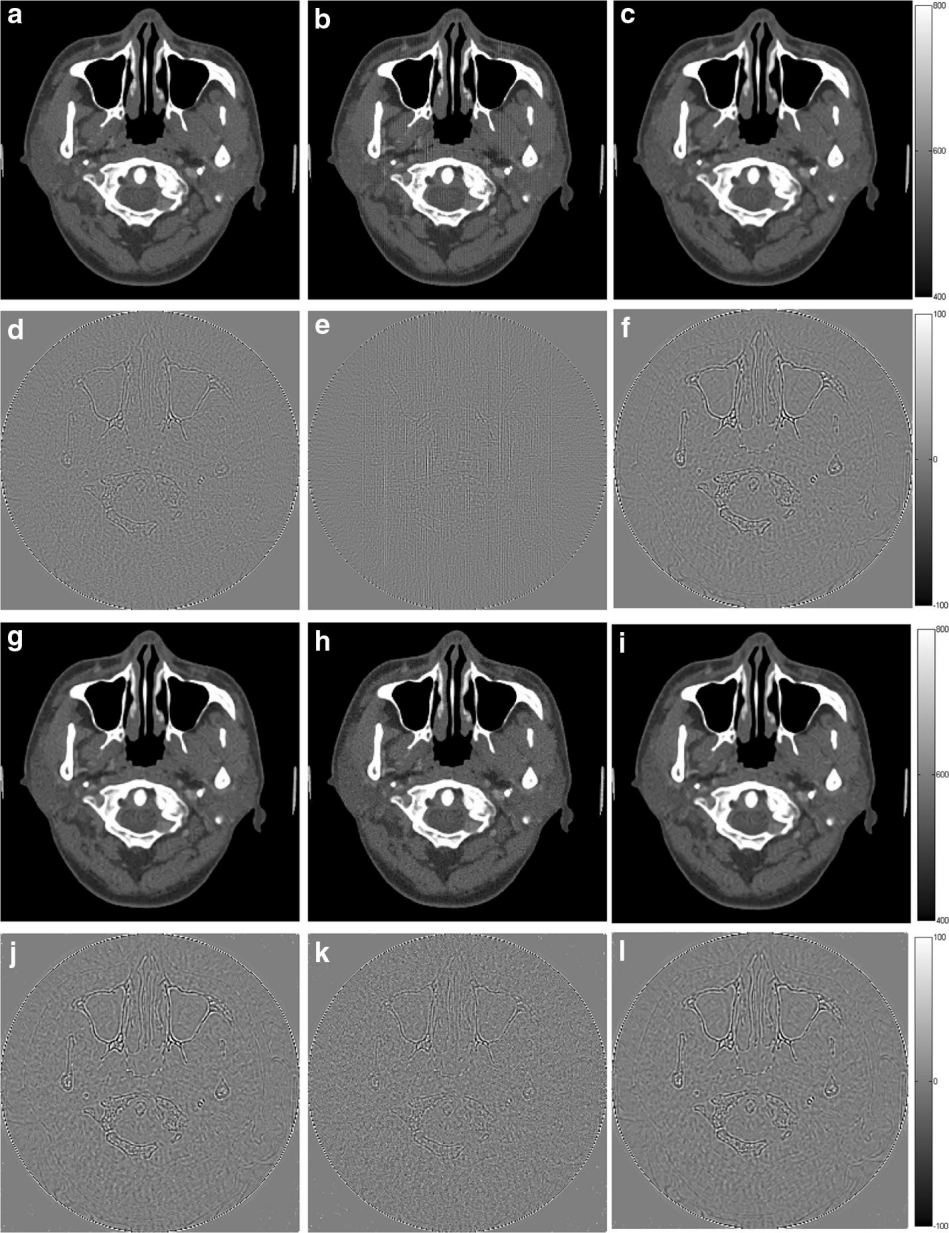
The reconstruction results of the human head slice by ADSIR with different regularization parameter. **a**–**c** The image reconstructed by 180 scanning views data with no noise; **d**–**f** the difference between the reconstructed image (**a**–**c**) and the original image (OI); **g**–**i** the image reconstructed by 180 scanning views data simulated by 2 million photons; **j**–**l** the difference between the reconstructed image (**g**–**i)** and the original image (OI). The *first* to *third columns* refer to the results with *λ*, 0.2*λ* and 5*λ* respectively

In this part, the rationality and influence of the regularization parameter are discussed in detail. To verify that the selections of this parameter in former text are rational and explore the influence to the results by other parameter values, the images are reconstructed by different regularization parameters. The phantom under simulation is the human head slice in Fig. 2. The projection data are 180 views with no noise, 180 views with noise simulated by 2 million photons. To each projection model, the regularization parameters are chosen as the one calculated by the fitting function (cited as *λ*), the one multiplied by 0.2 (cited as 0.2*λ*) and the one multiplied by 5 (cited as 5*λ*). The results of ADSIR are shown in Figs. 11 and 12. The RMSEs of the results are shown in Table 6.

**Table 6:** RMSEs (HU) of reconstructed images of different regularization parameters

Noise level	RMSE (HU)		
	*λ*	0.2*λ*	5*λ*
None	16.18	*10.25*	25.18
2 million photons	24.81	*17.85*	27.11

From the data in Table 6, we can see the images reconstructed by a bigger regularization parameter (5*λ*) will have larger RMSEs, which means the reconstructed images are influenced by the smoothing effect and fewer image details are reserved. When this parameter is set smaller (0.2*λ*), the RMSEs might be a little smaller than the ones reconstructed by the proper regularization parameter, such as the data as italics in Table 6 shows. However, the images in Figs. 11 and 12 (the second column) are ruined by noise or streak artifacts in different extents. Therefore, it is important to select the proper value of the regularization parameter for the DL-based methods and the simulations in this section prove that the selections in this article are rational.

## Discussion

The simulation results indicate that the proposed L_1_-DL algorithm is a useful and robust method for the sparse CT reconstruction. L_1_-DL utilizes more prior information of image sparsity than ADSIR benefited by the L_1_-norm DL regularization term. L_1_-DL is an improved method of ADSIR, and the simulation results demonstrate the image quality improvement of L_1_-DL than ADSIR.

By comparing the simulation results, another comparison algorithm, GPBB, is a little better than L_1_-DL in some situations when comparing the RMSEs. However, GPBB is not good at reserving the edge details and structures, especially when the sampling rate reduces further. It is claimed that L_1_-DL has a higher spatial resolution than GPBB.

In the simulation to explain the convergence rate of L_1_-DL, L_1_-DL stops at the 72th iteration while ADSIR stops at the 49th iteration and the time of one iteration is about 90 s. So it consumes about 108 and 74 min for the reconstruction of L_1_-DL and ADSIR. When it comes to the reconstruction processes of GPBB and SART with the same projection data, the time of one iteration of these two algorithm is about 1 s. Since GPBB and SART stops when the iteration number reaches 1000, the reconstruction time is about 17 min. In addition, the iteration process of GPBB and SART can be accelerated by GPU, so that the time can be reduced to less than a minute. Accelerating L_1_-DL, ADSIR and other DL-based methods is an important factor for the practical application of the DL-based methods.

## Conclusion

In this work, we propose to replace the L_2_-norm regularization term with the L_1_-norm one to improve image quality reconstructed by the DL-based method. The new objective function is optimized by the adaptive weighted L_2_-norm strategy, which is similar to the IRLS algorithm. By involving this modification, the proposed L_1_-DL algorithm behaves better than the existing DL-based method (ADSIR), and other two comparing algorithms. Experimental results show that the proposed algorithm can satisfy the demand of further radiation reduction in CT scanning since it needs fewer scanning data for high-quality recovery. In addition, the proposed algorithm retains the robust characteristic to the projection noise as a DL-based algorithm. Our future work will focus on two aspects. One of them is accelerating the DL-based methods to make the real time imaging with low-dose radiation possible. The other is looking for some possible strategies to utilize more prior information and further improve the image reconstruction result. For example, by utilizing a proper way to distinguish structural information and noise in the image, the DL regularization term can be designed based on the distinguishing results, which is a promising method to preserve more structural information and improve the image quality.

## Abbreviations

CT: computed tomography
DL: dictionary learning
IRLS: iteratively reweighted least squares
ADISR: adaptive dictionary based statistical iterative reconstruction
PLWS: penalized weighted least-squares
FBP: filtered backprojection algorithm
SART: simultaneous algebraic reconstruction technique
TV: total variation
DGT: discrete gradient transform
ASD-POCS: adaptive steepest descent projection onto convex sets
GPBB: gradient projection Barzilai Borwein
IRLS: iteratively reweighted least squares
SIR: statistical iterative reconstruction
ADSIR: adaptive dictionary based statistical iterative reconstruction
SNR: signal to noise ratios
K-SVD: k singular value decomposition
OMP: orthogonal matching pursuit
RMSE: root mean square error
